# Melatonin for Treatment-Seeking Alcohol Use Disorder patients with sleeping problems: A randomized clinical pilot trial

**DOI:** 10.1038/s41598-020-65166-y

**Published:** 2020-05-26

**Authors:** Marie N. S. Gendy, Dina Lagzdins, Jessika Schaman, Bernard Le Foll

**Affiliations:** 1 Translational Addiction Research Laboratory, Centre for Addiction and Mental Health, 33 Russell Street, Toronto, M5S 2S1 Ontario, Canada; 2 Alcohol Research and Treatment Clinic, Acute Care Program, CAMH, Toronto, M6J 1H4 Ontario, Canada; 3 Campbell Family Mental Health Research Institute, CAMH, 33 Russell Street, Toronto, M5S 2S1 Ontario, Canada; 4 Department of Family and Community Medicine, University of Toronto, 500 University Ave, Toronto, M5G 1V7 Ontario, Canada; 5 Department of Pharmacology, University of Toronto, Medical Science Building, 1 King’s College Cir, Toronto, M5S 1A8 Ontario, Canada; 6 Department of Psychiatry, Division of Brain and Therapeutics, University of Toronto, 250 College Street, Toronto, M5T 1R8 Ontario, Canada; 7 Institute of Medical Sciences, University of Toronto, 27 King’s College Cir, Toronto, M5S 1A8 Ontario, Canada

**Keywords:** Therapeutics, Translational research

## Abstract

A high percentage of subjects diagnosed with alcohol use disorder (AUD) suffer from sleeping difficulties. Lack of sleep could lead AUD patients to relapse or, sometimes, to suicide. Most of the currently prescribed medications to treat this complex problem retain a high risk of side effects and/or dependence. Therefore, the aim of the current clinical trial is to investigate the possibility of the use of a safer treatment, such as the natural health product melatonin, to treat alcohol-related sleeping problems. Sixty treatment-seeking AUD subjects were assigned to melatonin (5 mg) or placebo for 4 weeks of treatment. Change in sleeping quality which is the primary outcome of the study was assessed using the Pittsburgh sleep quality index (PSQI) scale. Linear mixed models were used to statistically analyze the difference in scores before and after 4 weeks of treatment. There was a reduction in the global PSQI score in both groups with no significant drug effect between groups. In conclusion, the use of melatonin (5 mg)/day didn’t differ from placebo in decreasing sleeping problems in a sample of AUD subjects after 4 weeks of treatment. However, higher doses are worth exploring in future research.

## Introduction

Over 70% of subjects diagnosed with alcohol use disorder (AUD) suffer from alcohol- induced sleep problems and some studies suggest that the percentage could reach up to 91%^1,2^. According to The Diagnostic and Statistical Manual of Mental Disorders 5th edition (DSM-5) disturbed sleep, or Sleep-Wake disorder, is further subdivided to insomnia, hyper- somnolence, circadian rhythm sleep-wake disorder, restless legs syndrome, narcolepsy, breathing-related sleep disorder, rapid eye movement (REM) disorder, non–rapid eye movement (NREM) sleep arousal disorders, nightmare disorder, and substance or medication-induced sleep disorder^2,3^.

The reasons explaining why AUD subjects would suffer from sleeping problems could be multifactorial: a genetic component, comorbid or preexisting depression or a general disturbance in the physiological wake-sleep circadian rhythm could all play a role^4^. Studies show that AUD subjects sleeping problems are present in all drinking stages i.e. active use, early and prolonged abstinence, and even during withdrawal^5,6^. Chronic alcohol use causes a decrease in the inhibitory response of GABA-A receptors accompanied by an increase in the excitatory activity of glutamate receptors^7^. Further, when alcohol effects wear off, AUD subjects experience an increase in sleep latency, or the time taken to fall asleep, with a decrease in total sleep duration, (circadian rhythm sleep-wake disorder); this may represent a mechanism by which tolerance to the hypnotic effects of alcohol could be explained^8^.

Polysomnography (PSG) studies examining sleep patterns in AUD found a persistent increase in light sleep or stage 1, a decrease in slow wave or deep sleep, an increase in vivid dreaming, and a disruption in REM sleep that could last for several months after sobriety^9–11^. Between 5 to 9 months of continuous abstinence is needed to normalize the time to fall asleep and sleep efficiency^12,13^, and more than a year is needed to restore normal sleep duration^12^. Disturbed sleep represents a special concern for AUD patients as it can lead to depression, cardiovascular complications, low quality of life, relapse, vehicle accidents and suicidal ideation^4,5,14–17^. Treated AUD subjects tend to relapse to alcohol to maintain a good sleep as a self-medication mechanism^18^. Therefore, no matter how successful AUD treatments are, the persistence of disturbed sleep represents a huge barrier to successful long term abstinence^19^.

It is not unusual for benzodiazepine receptor agonist medications to be prescribed to treat sleeping problems. Despite their well-known efficiency, benzodiazepine use is associated with impaired cognitive and psychomotor skills, increased risk of falls, dependence and abuse^14^. These side effects, including alcohol- induced risk of overdose and abuse potential, explains why addiction medicine practitioners avoid the use of benzodiazepines long after detoxification. Therefore, there is a real need for a safer treatment for sleeping problems, especially among patients suffering from substance use disorder (SUD)^4^.

Melatonin, (N-acetyl-5-methoxytryptamine), is an important hormone secreted by the pineal gland in response to darkness. It binds to melatonin receptors MT1 and MT2 in the suprachiasmatic nucleus and plays a fundamental role in the sleep-wake cycle^20^. Melatonin levels are usually low during the day and can reach a nocturnal peak up to 80–90 pg/ml, with some individual variability^21^. In normal individuals, the level of melatonin starts to increase before night time sleep, reaches its highest levels between 2:00 and 4:00 am then starts to decrease again around waking time. This peak melatonin surge is found to be blunted in AUD subjects^22,23^. In addition, chronic alcohol consumption alters melatonin production and functions, delays the nocturnal melatonin peak rise and decreases melatonin levels in AUD subjects^12,23,24^.

As a supplement melatonin has low oral bioavailability, a very short half-life (20–30 minutes), and is extensively metabolized by the liver enzymes^25^. On the market, melatonin is available as an over the counter dietary supplement as tablets, sublingual capsules and in liquid form. It is regulated by the FDA and Health Canada not as a medicinal drug but as a natural health product^26,27^. It is commonly used by people with sleep difficulties due to night shifts, jet lag, and restless leg syndrome or patients suffering from sleep-wake problems in general^28^. Most clinical trials show that melatonin significantly improves sleep quality, reduces sleep-onset latency period and the number of night-awakenings, as well as enhancing morning activity as assessed by validated sleep-wake questionnaires and/or PSG^29,30^.

Studies show that patients suffering from mood disorders, attention deficit hyperactivity disorder (ADHD), or schizophrenia, could use melatonin as an adjuvant treatment for their insomnia symptoms during acute phases of illness. In addition, the use of melatonin has proved to be helpful in the prevention of relapse among patients diagnosed with a stabilized psychiatric condition who complain of poor sleep quality^31^. It is not unusual to prescribe melatonin with or without other sleep medications for AUD subjects in medical institutions. However, there is currently no clear data showing efficacy of this approach. Therefore, this double- blinded randomized placebo-controlled pilot study was conducted to explore the efficacy of melatonin to treat sleeping problems in AUD subjects. To our knowledge, this is the first RCT using melatonin alone in AUD patients.

## Results

**Figure 1:**
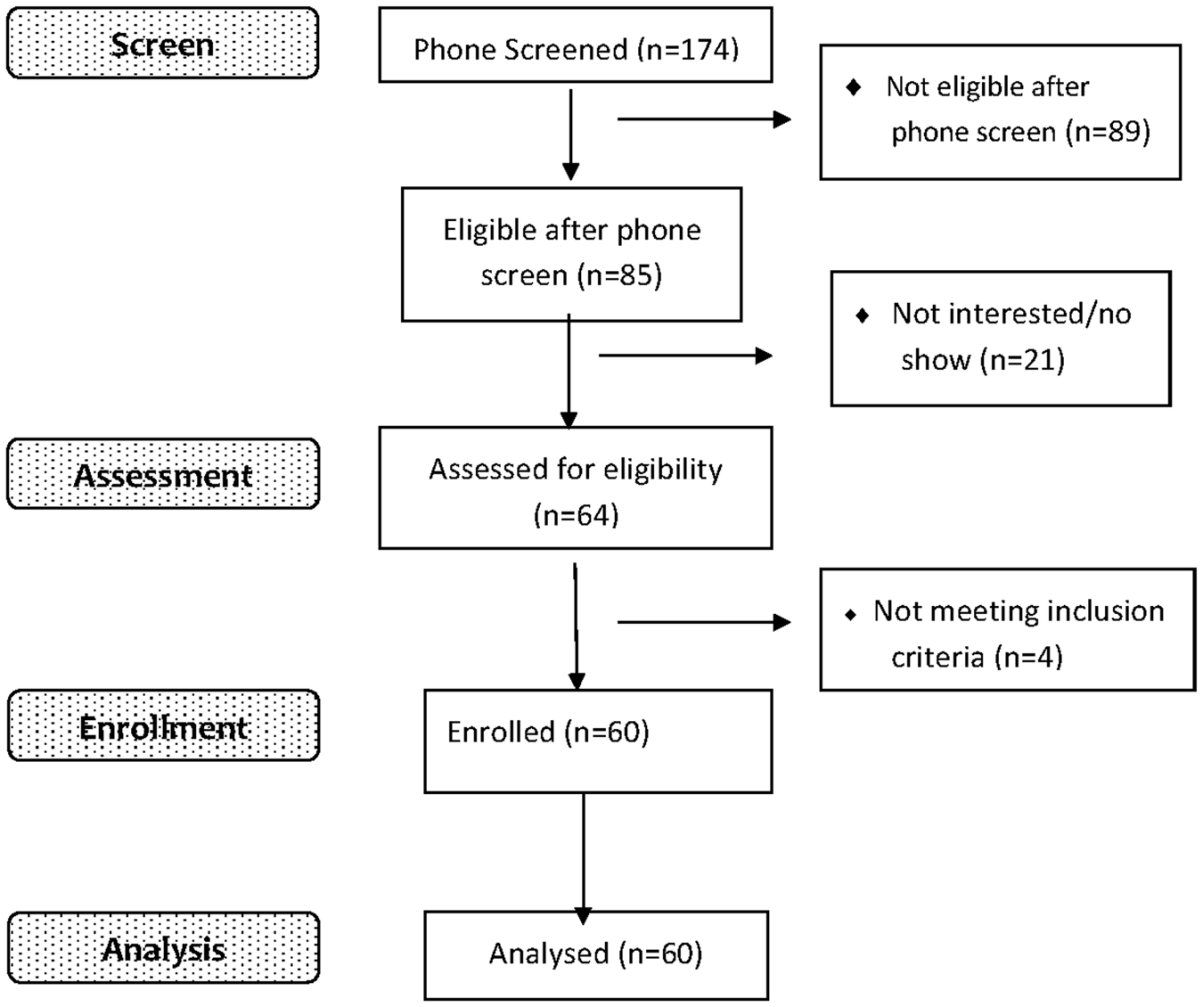
CONSORT Flow Chart.

Our sample included 60 treatment-seeking AUD participants as shown in Fig. 1. All subjects were randomly allocated into the melatonin group (n = 30) or placebo group (n = 30). There were 46 males (76.7%) and 14 females (23.3%). 75% of our sample were Caucasian, 3.3% were Black/African, 1.7% Asian and the rest of the sample were from mixed races. The mean Pittsburgh sleep quality index (PSQI) score (±SD) collected at baseline was 12.33 (2.93). Mean BDI and BAI (±SD) were 17.38 (8.44) and 15.43 (11.13) at baseline, respectively. The AUDIT score at baseline was 25.83 (±8.37). Detailed demographics for each group are shown in Table 1.

**Table 1:** Melatonin/ Placebo groups’ demographics.

Demographics	Melatonin	Placebo
	N = 30	N = 30
**Sex (n,%)**		
Male	23, 76.7%	23, 76.7%
Female	7, 23.3%	7, 23.3%
**Age range**		
19–25	0, 0.0%	1, 3.3%
26–40	10, 33.3%	11, 36.7%
41–60	16, 53.3%	17, 56.7%
>60	4, 13.3%	1, 3.3%
**Marital status**		
Single	14, 46.7%	17, 56.7%
Married	3, 10.0%	7, 23.3%
Divorced/separated	12, 40.0%	6, 20.0%
Widowed	1, 3.3%	0, 0.0%
**Employment**		
Full time (>35 hr/wk)	4, 13.3%	7, 23.3%
Short-term disability	3, 10.0%	3, 10.0%
Self-employed	3, 10.0%	3, 10.0%
Welfare	3, 10.0%	0, 0.0%
Student	1, 3.3%	1, 3.3%
Part time(<35 hr/wk)	1, 3.3%	4, 13.3%
Long-term disability	3, 10.0%	3, 10.0%
Not employed	7, 23.3%	10, 33.3%
Retired	5, 16.7%	0, 0.0%
**Highest completed education**		
Part of high school	0, 0.0%	2, 6.7%
High school	10, 33.3%	6, 20.0%
College	12, 40.0%	7, 23.3%
University	7, 23.3%	14, 46.7%
Graduate degree	1, 3.3%	1, 3.3%
**Ethnicity**		
Black or African American	1, 3.3%	1, 3.3%
Asian	0, 0.0%	1, 3.3%
Caucasian	24, 80.0%	21, 70.0%
Mixed races	5, 16.7%	7, 23.3%
Smokers	16, 53.3%	14, 46.7%
Non smokers	14, 46.7%	16, 53.3%
**AUD severity**		
Mild (2-3)	1, 3.3%	1, 3.3%
Moderate (4-5)	2, 6.7%	1, 3.3%
Severe (>6)	27, 90.0%	28, 93.3%
BDI mean at baseline (±SD)	16.30 (7.05)	18.47 (9.64)
BAI mean at baseline (±SD)	14.53 (10.08)	16.33 (12.20)
AUDIT score mean at baseline (±SD)	24.53 (8.67)	27.13 (7.99)
Global PSQI score mean baseline (±SD)	12.97 (2.28)	11.70 (3.37)

### Main findings

The results of pill counting and self-reported study medication logs were obtained from (n = 56) as 3 participants dropped out the study and 1 participant was excluded in the middle of the study due to not following the study procedures. The results showed 75% adherence to study medication (i.e. 75% took all the pills). The results of self-reported daily use of alcohol (n = 56) showed that 78.6% (n = 44) were successful in abstaining from alcohol during the 4 weeks of the study while 12 subjects (21.4%) consumed alcohol during the study. Among those who consumed alcohol during the study (n = 6) were from the placebo group and (n = 6) subjects were from the melatonin group. Melatonin was overall well tolerated, where mild to moderate side effects were reported and resolved over the course of the treatment. Irritability (n = 1), and weakness and dizziness (n = 1) were reported in the melatonin group while daytime sleepiness (n = 1), rash (n = 1) and vomiting (n = 1) were reported in the placebo group. No severe side effects were reported at all.

**Figure 2:**
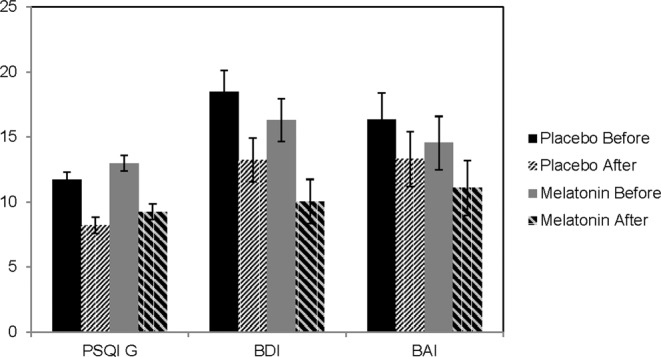
Main outcomes: PSQI, BDI, BAI scores before and after treatment expressed as Mean ± SD. All scores significantly decreased over time for the placebo group from baseline (black bars) to after treatment (2nd pattern bars). Also, the same scores decreased over time for the melatonin group from baseline (grey bars) to after treatment (4^th^pattern bars). No significant difference between groups was detected (*p* > 0.05).

**Figure 3:**
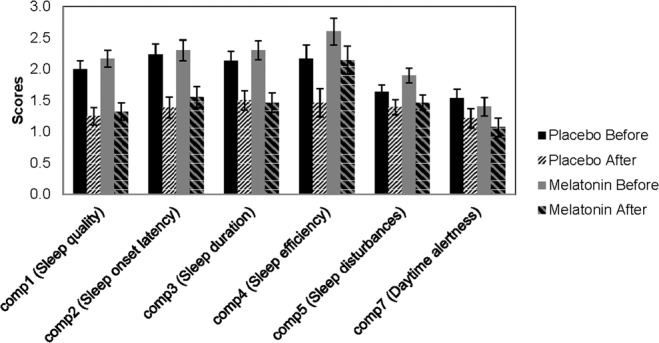
PSQI subscales, (components expressed as Mean ± SD), showed a significant decrease over time for the placebo group from baseline (black bars) to after treatment (2nd pattern bars). Also the scores showed a significant decrease over time for the melatonin group from baseline (grey bars) to after treatment (4^th^pattern bars). No significant difference between groups was detected (*p* > 0.05). Component # (6), which is the use of hypnotic sedative medication, is not shown in this figure as it was considered zero for this study.

Linear mixed models to analyze PSQI global score before and after treatment for both groups revealed a significant decrease over the period of the study for both treatments. However, there was no significant drug effect (Table 2; Fig. 2). PSQI subscales showed a significant time effect that was observed for both groups but no significant drug effect of melatonin was shown (Fig. 3). Anxiety and depression scores were collected before and after treatment using BAI and BDI, respectively. There was a significant decrease in BAI and BDI over time, but no significant difference between melatonin and placebo as shown in (Fig. 2; Table 2). This pilot study detected a small effect size of the results: Cohen’s h = 0.27 based on the difference in means of both groups as well as the standard deviation of the placebo group.

**Table 2:** A summary of the results showing PSQI Global scores, PSQI subscales, BDI and BAI.

Variable measure (mean + SEM)	Melatonin group N = 30		Placebo group N = 30		*F* Time*group	*P* value Time*group
	Baseline	Treatment	Baseline	Treatment		
PSQI global score	12.967 (0.594)	9.242 (0.611)	11.700 (0.594)	8.199 (0.611)	(1,56.338) = 0.058	0.81
Comp 1	2.167 (0.133)	1.321(0.138)	2.0 (0.133)	1.250 (0.138)	(1,112) = 0.124	0.726
Comp 2	2.300 (0.165)	1.551 (0.170)	2.233 (0.165)	1.387 (0.170)	(1,55.658) = 0.130	0.720
Comp 3	2.300 (0.150)	1.464 (0.155)	2.133 (0.150)	1.500 (0.155)	(1,112) = 0.439	0.509
Comp 4	2.600 (0.217)	2.143 (0.224)	2.167 (0.217)	1.464 (0.224)	(1,112) = 0.309	0.579
Comp 5	1.900 (0.116)	1.464 (0.120)	1.633 (0.116)	1.393 (0.120)	(1,112) = 0.682	0.411
Comp 7	1.400 (0.146)	1.071 (0.151)	1.533 (0.146)	1.214 (0.151)	(1,112) = 0.001	0.974
BDI	16.300 (1.653)	10.042 (1.690)	18.467 (1.653)	13.222 (1.690)	(1,56.368) = 0.226	0.637
BAI	14.533 (2.050)	11.074 (2.097)	16.333 (2.050)	13.295 (2.097)	(1,55.934) = 0.024	0.877

A list of the concomitant medications and concurrent medical conditions assessed at baseline for all the subjects by a physician is shown in Table 3. Only (17.9%) of participants were not using anti-craving and/or antidepressant medications that would affect sleep. Further analyses of PSQI were conducted excluding all subjects using anti-craving and/or antidepressant medications. PSQI score showed a significant decrease over time with no significant melatonin effect; [*F* (1, 16 = 0.629); *p* = 0.439]. Also, secondary analysis for the subjects who maintained complete abstinence from alcohol (78.6%) during the 4 weeks of the study showed a significant time effect where PSQI decreased over time with no drug effect for melatonin [*F* (1, 86 = 0.031); *p* = 0.861].

**Table 3:** Concomitant medications and medical conditions at baseline.

	Melatonin (n, %)	Placebo (n, %)
**Medications**		
Anticoagulants	(2, 6.7%)	(0, 0.0%)
Anticonvulsants	(4, 13.3%)	(1, 3.3%)
Blood pressure medications	(5, 16.7%)	(3, 10.0%)
Sedatives or hypnotics	(0, 0.0%)	(0, 0.0%)
Psychotropic medications	(20, 66.7%)	(19, 63.3%)
Steroids	(0, 0.0%)	(1, 3.3%)
Others	(12, 40.0%)	(16, 53.3%)
None	(5, 16.7%)	(4, 13.3%)
**Medical condition**		
Asthma	(4, 13.3%)	(2, 6.7%)
Cardiovascular disease	(6, 20.0%)	(1, 3.3%)
Chronic kidney disease	(0, 0.0%)	(0, 0.0%)
Depression	(20, 66.7%)	(19, 63.3%)
Diabetes	(0, 0.0%)	(0, 0.0%)
Liver disease	(0, 0.0%)	(1, 3.3%)
Migraine	(1, 3.3%)	(3, 10.0%)
Seizure disorders	(1, 3.3%)	(4, 13.3%)
Hormonal disease	(0, 0.0%)	(0, 0.0%)
None	(9, 30.0%)	(7, 23.3%)

## Discussion

The current RCT studied the effect of melatonin 5 mg on sleeping problems in 60 treatment- seeking AUD subjects versus placebo for 4 weeks. PSQI global score and subscales were significantly decreased at the end of the treatment period. Nevertheless, there was no significant drug effect. Further analyses of mood showed a significant time effect where anxiety and depression scores decreased significantly after 4 weeks. However, no drug effect was observed.

Controversy about the effect of a drug is not unusual in the scientific literature. Although some previous studies showed promising effects of melatonin, other placebo-controlled trials showed different results. Our results are consistent with a study among patients diagnosed with Alzheimer’s disease (AD) where melatonin (10 mg) didn’t improve sleep quality vs placebo^32^. Another study among patients diagnosed with dementia didn’t show a significant effect of melatonin (6 mg) on sleep after 2 weeks of treatment^33^. Further, pooled data from three different RCTs in 209 AD patients suffering from sleep disturbances revealed no significant difference between melatonin and placebo^34^. One interpretation is that these results could be explained by a weakening of the effect of melatonin due to the presence of a mental illness that could interfere with the circadian rhythm^35,36^.

On the other hand, PSQI index improved significantly after 4 weeks of melatonin (3 mg) treatment compared to placebo in a study of 18 patients diagnosed with Parkinson’s disease and sleeping disturbances,. Nevertheless, PSG was not improved. The authors suggested that this discrepancy between PSQI and PSG was due to the small sample size and the complexity and variations of the PSG observations^37^.

In this study, all subjects were asked to maintain 2–3 weeks of alcohol abstinence before the start of the medication to ensure their adherence to the 4 weeks of abstinence during the study. Abstinence at baseline was verified by Time Line Follow Back (TLFB) for the last 2 weeks. Our sample was in early recovery from alcohol (2 to 8 weeks) at baseline. Our findings are consistent with other studies showing that the persistence of sleep disturbance during early recovery, including increased sleep onset latency, decreased sleep duration, and low sleep efficiency, contributes to the late withdrawal symptoms or the protracted abstinence and the CNS hyper-excitability found in AUD subjects after several weeks of abstinence^38–41^.

Initial insomnia, or difficulties initiating sleep (PSQI component # 2), and middle insomnia, or interrupted sleep, are the 2 main sleeping problems experienced by individuals with AUD^1,42^. Therefore, in the current study, it was hypothesized that AUD subjects would benefit from the use of melatonin, as a previous meta-analysis of 15 studies in healthy subjects(n = 1683) demonstrated that melatonin specifically reduced sleep onset latency by 3.9 minutes and enhanced overall sleep duration by 13.7 minutes^43^. However, in our study there was a decrease in PSQI components over time but that decrease wasn’t accompanied by a significant drug effect. This discrepancy could be due to the fact that participants in the other studies included in the meta-analysis didn’t have any medical or psychological comorbidity that could interfere with the action of melatonin on sleep. Another interpretation is that the damaging effects of alcohol on the wake-sleep cycle could not be reversed with only 4 weeks of treatment, since studies suggest that damage to sleep quality could take more than 5 to 9 months of sobriety to be improved^44,45^ and it could take up to 14 months to restore normal sleep duration^13^. This could partially explain the negative results in the present study taking into consideration the short period of abstinence. In this regard, we performed secondary analyses to examine alcohol abstinence effects on sleep. The results for subjects who maintained complete abstinence from alcohol (n = 44, 78.6%) during the 4 weeks of the study showed a significant time effect, whereby PSQI decreased over time with no drug effect (P > 0.05) (data not shown).

To our knowledge this is the first RCT using melatonin (5 mg) taken orally 1 hour before bed time for 4 weeks of treatment in AUD subjects. Our results were not consistent with the results of open-label studies that explored MT1 and MT2 synthetic agonists in AUD subjects. Ramelteon (8 mg) was used 30 minutes before bed time in an open-label study with 5 AUD participants for 4 weeks and showed a decrease in signs of insomnia according to the Insomnia Severity Index and a sleep diary^45^. Another open-label study showed that Agomelatine, an MT1and MT2 agonist, led to a significant improvement in PSQI score in 9 AUD subjects after 6 weeks of treatment^46^. However, none of these trials had a control group using placebo which therefore provides weak statistical evidence.

In our sample of patients the average AUDIT score at baseline was 25.83 (±8.37) with the majority meeting severe dependence criteria. PSQI score was high in both groups. A positive correlation between AUD severity and sleep disturbance measured by PSQI(perceived sleep quality factor, and daily disturbance factor) in non-treatment-seeking AUD subjects (N = 295) was shown previously, where higher PSQI scores were correlated with greater severity of AUD.

We hypothesized that melatonin would enhance mood and decrease anxiety more than placebo based on previous research showing that melatonin has significant anxiolytic effects^47^. Moreover, agomelatine, a melatonin receptor agonist, is used as anti-depressant in some countries. However, while the results of the present study showed an improvement in mood and anxiety over time, there was no significant drug effect on mood scores. These results are consistent with pooled data from eight clinical trials in patients diagnosed with mood disorders, including depression and bipolar disorder, which showed no significant mood improvement following treatment with melatonin^48^. This review concluded that although the use of melatonin alone did not appear to improve depressive or anxiety symptoms, a synergistic effect may be observed when melatonin is given as add-on therapy combined with other anxiolytic or antidepressant medication.

The current data, showing an improvement in mood over the course of the study irrespective of treatment condition, may reflect enhancements in sleep quality as a result of increasing duration of alcohol abstinence, which in turn may have had a positive effect on decreasing depression and anxiety scores.

Cognitive-behavioral therapy (CBT)-I, which includes cognitive therapy, sleep hygiene, sleep restriction and stimulus control^49^, is recommended as a 1^st^ line treatment for insomnia in general populations^50^ and is thought to be effective in treating sleep difficulties in AUD subjects. However, the long therapy duration, commitment and the lack of highly trained professionals could limit CBT-I use in practice^51^. In this study, CBT sessions were not applied, however all subjects were provided with a sleep hygiene form during the medication visit as a helping tool to be used during the study^51^. Practicing sleep hygiene techniques is only likely to be successful if used as part of a daily routine, and establishing healthy sleep habits could take a long time to be achieved^52,53^.

While melatonin is a natural health product, Ramelteon and Tazimelteon, approved by the FDA in 2005 and 2014, respectively, are two synthetic melatonin agonists used for sleep problems. Both are characterized by a higher half-life compared to melatonin; Ramelteon T1/2 is 1 to2.6 hours and Tasimelteon T1/2 is 1.3 to 3.7 hours. *In vitro* research for receptor binding assays showed that Ramelteon had a greater selectivity and affinity for both MT1 and MT2 receptors and a significantly higher dissociation constant from MT receptor binding sites. Also it showed lesser affinity than melatonin to MT3, the melatonin related enzyme Quinone reductase. Clinically, Ramelteon proved to be 10 times more potent than melatonin at enhancing sleep quality. For instance, it showed better results in shortening sleep onset latency and increasing sleep duration compared to melatonin. Although studies on Tasimelteon have shown that it is a melatonin-receptor agonist at the MT1 and MT2 receptors, it shows higher affinity for the MT2 receptor, which explains its use for non-24 hour sleep-wake disorder in blind subjects. Nevertheless, both Ramelteon and Tazimelteon are much more expensive than melatonin^54,55^. In this study, natural melatonin was used, although it is worth investigating synthetic melatonin agonists in future research.

The negative findings of the present study are not surprising as the clinical and preclinical findings are still very controversial, but it now seems clear that melatonin likely acts only in helping to fall asleep rather than in increasing sleep time. This could be explained by the very short half-life of melatonin and also by the contrasting/complementary role of melatonin receptors on sleep phases. Preclinical studies showed that MT1 selective stimulation increased rapid eye movement (REM) sleep while MT2 receptors increased non-rapid eye movement (NREM) sleep highlighting an opposite role of both receptors^56^. Further research investigating melatonergic receptors and different ligands is needed in order to clarify the specific role of each ligand.

There are some limitations of the current study. For instance, only one dose (5 mg) of fast dissolving melatonin tablets was used. It would therefore be worthwhile to explore higher doses and an extended release formulation of melatonin. In addition, in this study melatonin was used as single treatment, while in practice melatonin is prescribed as an adjuvant drug to be taken in combination with other sleeping pills. Therefore, it is worth exploring the different effects on sleep quality when melatonin is used alone versus in conjunction with other sleep aids.

Further limitations of the study are that medication adherence was verified by self-report and pill count at the end of the study, whereas weekly verification may have reduced recall bias; the timing of melatonin administration was not objectively assessed - future research could make use of smart pill bottle technology to record the time of the pill taken; and finally sleep was not objectively assessed and therefore future research would benefit from the use of daily sleep diaries, actigraphy or polysomnography, and assessment of blood levels of melatonin.

In conclusion, it is quite common for AUD patients to relapse to drinking alcohol to self-medicate their sleeping problems^15^. Therefore, it is worth conducting further studies with melatonin using different doses and meticulous follow up in order to find a safe strategy for the treatment of alcohol-related sleeping problems to prevent relapse. The results of the current study showed no significant effect of 4 weeks oral melatonin (5 mg) on attenuation of sleep problems in treatment-seeking AUD subjects.

## Methods

### Study design

This study is a double blind randomized placebo-controlled trial with two arms. Sixty outpatient treatment-seeking subjects diagnosed with AUD and suffering from sleeping problems were recruited from Addiction Medical Service clinic and other clinics at the Center for Addiction and Mental Health (CAMH) using study posters and staff referrals. Recruitment started January 2017 and the study was closed January 2019. Informed consent was obtained from all individual participants included in the study. Sleeping quality, which is the primary outcome of the study, was assessed using PSQI score which is a validated questionnaire formed of 7 components^57^. The PSQI is widely used by sleep specialists to evaluate sleep disturbance as a diagnostic tool for sleep problems, and is used in research for assessment of treatment outcomes^37,46^. The scale is formed of 19 self-rated questions and another 5 questions answered by the bed partner or roommate if present. The scoring of the PSQI is dependent on the 19 self-rated questions, the 5 other questions are used for clinical evaluation purposes only. The 7 components of the questionnaires are each scored on a 0–3 scale. The sum of all 7 component scores forms the global PSQI score, which ranges from 0-21; the higher the score the worse the sleep quality. For this project, component # 6, which records the number of times a sleeping aid was used during the past month, was scored as zero because this overlapped with one of the exclusion criteria. Usually, a PSQI score higher than 5 indicate a sleep problem^57^. The inclusion criteria for this study were: aged19 years or older, PSQI > 5, meet DSM-5criteria for AUD, not taking a benzodiazepine receptor agonist or any other sleeping pills during the past month. Any subject not fulfilling the inclusion criteria or pregnant, (as verified by a urine test), were excluded from the study. All subjects were assigned randomly to either melatonin (5 mg) or placebo arms. The melatonin tablets used were: Nature’s bounty Melatonin (5 mg), NPN: 80033974, fast dissolving tablets. Study approvals were obtained from The Center for Addiction and Mental Health Research Ethics Board (REB) following all relevant guidelines and regulations (Protocol ID: 099-2016). The study was registered with the clinical studies database ClinialTrials.gov (NCT03043443, registration date: 06/02/2017).

Demographics were collected from all subjects at baseline, including: contact information, concomitant medication, Time Line Follow Back (TLFB)^58^ for the past 2 weeks regarding the use of nicotine, alcohol drinks/day, caffeine, cannabis and other substances. A single alcohol drink serving contains about 14 grams of ethanol or “pure” alcohol which could be 12 oz. of beer (about 5% alcohol), 8-9 oz. of malt liquor (about 7% alcohol), 1.5 oz. of hard liquor (about 40% alcohol), or 5 oz. of wine (about 12% alcohol). Fagerstrom test for nicotine dependence (FTND)^58^, Beck Depression Inventory (BDI)^59^, Beck Anxiety Inventory (BAI)^60^, AUD criteria according to DSM-5^61^, and Alcohol use disorder identification test (AUDIT)^62–63^ were also collected at baseline.

After verifying their eligibility, participants were randomized. Subsequently they picked up the medication (Melatonin or placebo), provided in a blister pack with instructions and a sleep hygiene document, and were instructed to take 1 pill every night 1 hour before bedtime for 4 weeks. PSQI score was measured again after 4 weeks of treatment. An online survey was sent after 2 weeks and after 4 weeks of treatment to monitor side effects along with other questionnaires (TLFB, BDI, BAI). All participants were asked to bring back the medication blister pack to do a pill count and check all the missing pills. In this study, participants were compensated for their participation over the course of the study with $85; $20 was provided at the assessment visit and the remainder of the compensation was provided after completion of all the visits and the surveys. All study medications (Melatonin and placebo) were dispensed by the CAMH pharmacy that was also responsible for all randomization as well as blinding procedures (for both researchers and subjects).

The sample size was calculated based on Mixed Effect Models and significance tests, where all tests were two-sided, using a confidence level of 0.05 with power fixed at 80%.

### Data analyses

Our primary outcome measure was sleeping problems measured using the PSQI global score at baseline and after 4 weeks of treatment. Secondary outcome measures included the subscales of PSQI which are: (1) subjective quality of sleep; (2) sleep onset latency; (3) sleep duration; (4) sleep efficiency; (5) presence of sleep disturbances; and (7) presence of daytime disturbances, as an indication of daytime alertness. Also, BDI and BAI scores before and after treatment were considered as secondary outcomes. Linear mixed models with subjects as random effects were used to analyze the outcomes. An interaction between time (pre/post treatment) and treatment groups (melatonin/placebo) was used to investigate difference in change in PSQI global score as well as sub-scores, BDI and BAI scores between study groups. All analyses were conducted using SPSS v.24. Associations with p-values of less than 0.05 were considered statistically significant, and all tests were 2-sided. Both sex and age were controlled for in the analysis to limit confounding by these variables.

### Study procedures

All the study procedures and methods as well as the study approvals were obtained from The Center for Addiction and Mental Health Research Ethics Board (REB) following all relevant guidelines and regulations (Protocol ID: 099-2016). Also, a detailed description of the study is found in ClinialTrial.gov website ID# NCT03043443/ (registration date: 06/02/2017).

## Data Availability

All additional data, research protocol, and information on materials used in this investigation will be made readily available upon request as allowed by the governing review boards of the involved research institutions.
